# Planning a holistic summative eHealth evaluation in an interdisciplinary and multi-national setting: a case study and propositions for guideline development

**DOI:** 10.1186/s12911-021-01399-9

**Published:** 2021-02-17

**Authors:** Monika Jurkeviciute, Amia Enam, Johanna Torres-Bonilla, Henrik Eriksson

**Affiliations:** 1 Centre for Healthcare Improvement (CHI), Chalmers University of Technology, Vera Sandbergs allé 8, 41296 Gothenburg, Sweden; 2 Present Address: NTNU, Sentralbygg 1, Gløshaugen, Alfred Getz vei 3, Trondheim, Norway; 3 Pasaje De Los Virreyes N27-35 y Calle Selva Alegre, 170520 Quito, Ecuador

**Keywords:** eHealth, Evaluation, Assessment, Evaluation planning, Guidelines, Standard, Evaluation quality, Stakeholder

## Abstract

**Background:**

Summative eHealth evaluations frequently lack quality, which affects the generalizability of the evidence, and its use in practice and further research. To guarantee quality, a number of activities are recommended in the guidelines for evaluation planning. This study aimed to examine a case of an eHealth evaluation planning in a multi-national and interdisciplinary setting and to provide recommendations for eHealth evaluation planning guidelines.

**Methods:**

An empirical eHealth evaluation process was developed through a case study. The empirical process was compared with selected guidelines for eHealth evaluation planning using a pattern-matching technique.

**Results:**

Planning in the interdisciplinary and multi-national team demanded extensive negotiation and alignment to support the future use of the evidence created. The evaluation planning guidelines did not provide specific strategies for different set-ups of the evaluation teams. Further, they did not address important aspects of quality evaluation, such as feasibility analysis of the outcome measures and data collection, monitoring of data quality, and consideration of the methods and measures employed in similar evaluations.

**Conclusions:**

Activities to prevent quality problems need to be incorporated in the guidelines for evaluation planning. Additionally, evaluators could benefit from guidance in evaluation planning related to the different set-ups of the evaluation teams.

## Background

eHealth, an information and communication technology that supports healthcare provision [1], is being piloted increasingly in healthcare settings, to understand whether and how it could improve health care. Often, expensive summative evaluations are conducted to assess the effectiveness and worth of eHealth in a particular setting, to develop knowledge, and to generate evidence for decision-making regarding investment in eHealth. Emphasis on such an evaluation is growing and it is reinforced by various organizations, such as the World Health Organization (WHO), European Society of Cardiology, International Medical Informatics Association (IMIA), and others [2–4]. However, quality of eHealth evaluations is often insufficient, with problems stemming from the application of non-scientific methods and non-standardized measures, insufficient attention to data collection and its feasibility, too large or unrealistic scope, mismatch between the technology and measures, and wrong assumptions about data quality [5, 6]. The growing number of eHealth evaluation studies does not compensate for the limited quality in several studies [7], and it becomes challenging to compare evidence between relevant studies to continuously learn in organizations and research communities, and to generalize knowledge [8].

Some scholars argue that social, economic, and political circumstances can affect robustness of the evaluation and consequently decision-making regarding the deployment of eHealth in routine health care [9]. The social aspects of eHealth evaluation teams are also becoming increasingly important because more evaluations are conducted in interdisciplinary and multi-national set-ups [10]. Previous research has highlighted several benefits of such set-ups. Interdisciplinary evaluation can help to prevent poor understanding of the context, and organizational and social issues [11], to reveal new evaluation questions [12], and to produce better insights [13]. However, the collaborating parties need to align their goals, agendas, and interests [10], and to build consensus [14].

In previous research, issues of quality in eHealth evaluation have been addressed through the creation of different guidelines and frameworks (e.g., [15]), by mapping relevant theories on technology and evaluation to the eHealth life cycle to detect essential themes for evaluation (e.g., [1]), by writing viewpoint articles (e.g.[4, 9, 10], or by analyzing the lessons learned from eHealth evaluation through systematic reviews or case studies (e.g., [16–19]). Apart from the specific guidelines and frameworks that address the planning or reporting stages of evaluation, most studies assume a holistic approach, and they do not focus on any specific part of the evaluation process.

In the present study, we focused on the planning stage of eHealth evaluation. Previous research has addressed this stage from a methodological perspective. Several guidelines have been developed, including the Guidelines for Internet Intervention Research [8], Design and evaluation guidelines for mental health technologies [20], Model for Assessment of Telemedicine Applications (MAST) [15], Health Information Technology Evaluation Toolkit (AHRQ) [21], and Guideline for Good Evaluation Practice in Health Informatics (GEP-HI) [22]. Meanwhile, the planning stage of the eHealth evaluation has not been addressed empirically. However, opinions regarding the value of planning are conflicting. While some scholars believe in thorough planning [22, 23], others advocate for an emergent and flexible approach, and doubt if evaluation can and should be planned in advance [10]. To address this debate, we set out to study the planning activities empirically in a multi-national and interdisciplinary setting, and to examine eHealth evaluation planning guidelines. In health research, scholars and professional societies have emphasized the significance of improving existing standards and assessing their effectiveness for particular contexts (e.g. [4, 24–27]). For the present study, we sought guidelines that discuss the process of eHealth evaluation planning and provide a step-by-step guidance. Accordingly, the AHRQ and GEP-HI guidelines were found the most suitable.

The research objective of this study was to examine the eHealth evaluation planning process in a multi-national and interdisciplinary setting and to provide recommendations for the development of eHealth evaluation planning guidelines. To achieve this research objective the following two research questions guided our work:

1. How can the eHealth evaluation planning process be described in a multi-national and interdisciplinary setting?
2. How can existing eHealth evaluation planning guidelines be improved to support eHealth evaluations?

Our intention with the research question 1 was to present a description of an eHealth evaluation planning process which could be used as a foundation to accomplish the research objective. In the research question 2 we were interested to understand the possible match between the guidelines and our description of the planning process, and by doing that to provide recommendations in accordance with the research objective. The remainder of this article is organized as follows. Methods section describes the methodology used to develop the empirical eHealth evaluation planning process and to compare it with GEP-HI and AHRQ guidelines. Results section presents the empirical process diagram and description, and the key findings from the aforementioned comparison. Reflections on eHealth evaluation planning in practice and recommendations for the development of guidelines are discussed in "Discussion" section.

## Methods

### Research setting

The empirical setting for this study was a multi-national European Union project “Digital Environment for Cognitive Inclusion (hereinafter called DECI) conducted in 2015–2018. The objective of DECI was to improve the quality of life and increase the independence of elderly individuals diagnosed with mild cognitive impairment or mild dementia. DECI aimed to provide the following eHealth services in a home environment: (1) an integrated care platform for communication between different stakeholders, (2) indoor sensors and a wearable watch for monitoring patients’ activity, and (3) physical and cognitive web-based training programs for patients. The solutions were applied in four countries. The business lines of the partner organizations were different, and they comprised medical, technological, and scientific aspects. The benefit of studying a single case like DECI is the opportunity for an in-depth description and explanation of the complexities of eHealth evaluation and its context, which may not be captured by other methods [28].

### Data collection

Multiple sources were used to extract data for the empirical evaluation planning process in DECI. The evaluation planning period was between September 2015 and September 2017, and the data collected consisted of all the e-mail correspondence available to the authors (*n* = 262) [29], which were related to evaluation planning, electronic versions of the developing evaluation plan (*n* = 32), and minutes from the meetings and calls of the consortium members (*n* = 8). Since the authors were in charge of the evaluation planning activities in the project, all related e-mail correspondence and materials were available.

### Data analysis

#### Evaluation planning process of DECI

The empirical data were organized in a chronological set of 301 information records by one researcher, to reflect the activities performed in connection with evaluation planning (hereinafter referred to as *activities*). To extract a meaningful overview of the process of evaluation planning, the 301 activities were aggregated using codes that helped organize, aggregate, and link the data [30]. In this case, the *codes* (*n* = 21) reflected summative features in the activities [31]. In order to create a more concrete process view, the codes were reviewed and aggregated to higher-level categories (*n* = 13) when the activities reflected by a code would be part of a bigger task (category). The codes and categories were subjectively defined by the first author. The reasoning used in the creation of the codes and their aggregation into the categories were thoroughly documented [31]. Two other authors examined the material and provided insights and suggestions for changes in the codes and categories or their use. Discrepancies were resolved and documented.

Thereafter, DECI evaluation planning activities were analyzed by three-month periods (seven periods in total). The categories became steps in the evaluation planning process map. To identify the sequential place of each category in the process, we examined the time stamps of the codes within each category. The place was determined by the highest rate of appearance of the related codes within the periods. The result was a 13-step map of the DECI evaluation planning process. Lastly, the steps were separated into the following two phases of evaluation planning: analyzing and planning. Finally, the steps were grouped under a certain phase according to similarities in the objectives of the activities carried out in a given step.

#### Comparison between the DECI case and other guidelines

It should be noted that the GEP-HI was not considered in its full scope for this analysis, since only the first four of its phases address the activities of planning an evaluation (preliminary outline, study design, operationalization of methods, and project planning).

The GEP-HI and AHRQ guidelines were compared to the DECI process using a pattern-matching technique [32], whereby a theoretical pattern is compared with an observed pattern. The purpose and activities of the steps in the guidelines were compared to those in the DECI process. The steps sharing a similar purpose and activities were grouped as a “match,” and those with no similarities in activities or purpose were grouped as “no match.” Three authors conducted the analysis separately. Their results were then compared and discussed, and the differences were resolved through consensus. During the comparison, we emphasized on the content of every step in the guidelines. While some titles of the steps may have looked similar, it was deemed important to verify the similarity of the content, which sometimes led to finding different interpretations of the steps.

## Results

### How can an eHealth evaluation planning process be described in a multi-national and interdisciplinary setting?

**Fig. 1:**
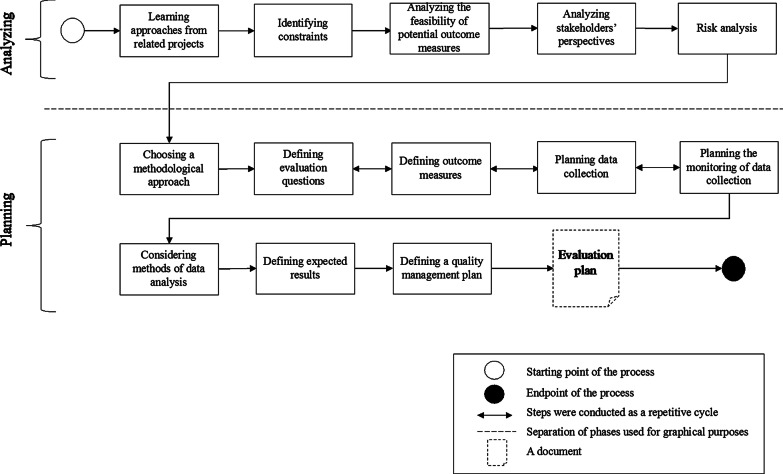
Evaluation planning process of DECI

The DECI evaluation planning process was outlined in two phases, analyzing and planning (see the process diagram presented in Fig. 1).

All activities concerning gathering knowledge and information regarding the project took place during the analyzing phase. These steps are described in Table 1.

**Table 1:** Steps in the “Analyzing” phase of the DECI evaluation planning

Step	Purpose	Step description
Learning approaches from related projects	To increase the generalizability of the DECI results and to support learning across similar studies	The contexts, methodologies, and lessons learned in similar studies were explored. Published study protocols and reports were reviewed, and researchers were contacted and their advice was sought. Published lessons learned from related projects were examined for applicability to DECI
Identifying constraints	To identify constraints and to understand how they affect the evaluation	To gain an in-depth understanding, different constraints (e.g., limitations due to the effect of patient diagnosis on feasible methods) were discussed between the partners. Potential impact on the evaluation was assessed
Analyzing the feasibility of potential outcome measures	To select feasible and commonly acceptable outcome measures	To select measures to be assessed in the project, a survey was conducted among the partners to understand the standard measurement practices in the project locations. A list of potential outcome measures was created based on the measures used by the partners and those used in similar studies. Consensus was sought on which outcome measures would be commonly used for evaluation of DECI
Analyzing stakeholders’ perspectives	To understand how different partners approach the project and the evaluation	First, interviews with the project partners were conducted to understand their perspectives, goals, and expectations related to the project. To create awareness of the perspectives of different partners and to facilitate negotiations in the consortium, a workshop was conducted to expose and align differences in these perspectives
Risk analysis	To identify the key threats to the project and the evaluation	Interviews with project partners were conducted to identify key risks, which were then aggregated and a risk management plan was outlined

Table 1. Steps in the “Analyzing” phase of the DECI evaluation planning.

The Planning phase provided a concrete shape to the evaluation plan. During this phase, the methodology was chosen and building blocks of the evaluation plan were created, e.g., evaluation questions and measures, and data collection was planned. Table 2 provides a description of the steps involved in the planning phase.

**Table 2:** Steps in the “Planning” phase of the DECI evaluation planning

Step	Purpose	Step description
Choosing a methodological approach	To select a methodological approach for the evaluation	Members of the interdisciplinary research consortium prescribed to different research traditions; therefore, the methodological approach was a subject for discussion. Quantitative and mixed-method approaches were considered. The mixed-method approach was selected
Defining evaluation questions	To define the research questions and endpoints	The questions of evaluation defined in the project contract were revised through recurring communications among partners. Owing to the interdisciplinary nature of the consortium, differences in the preferred endpoints and evaluation questions were resolved and a final list of research questions, and primary and secondary endpoints was created
Defining outcome measures	To define the outcome measures that will help assess the research questions and endpoints	Common outcome measures among all project locations were preferred. After rounds of negotiations, common outcome measures were selected. Means of acquiring data on the measures were defined, which resulted in several standard quantitative measures to assess health outcomes, tailor-made surveys on willingness-to-pay and physical activity levels, qualitative interview protocols to cover perceptions of patients and staff, and MS Excel files for the collection of organizational process data
Planning data collection	To make arrangements for data collection	Each partner’s technical and organizational possibilities for data collection were studied, taking into consideration data security principles and privacy laws. A schedule, format, and medium of data collection and its storage were planned
Planning the monitoring of data collection	To ensure the quality of the data collected	A template for monthly reporting of the data collected was created and shared with the partners responsible for the data collection
Considering methods of data analysis	To plan the data analysis	Feasible methods of data analysis were discussed among the project partners. Resources, timeplan, and competences needed were considered. Responsibilities for different parts in the data analysis were shared among different partners
Defining expected results	To define the expected outcomes of the intervention	Related studies and the stakeholders’ opinions were taken into account when setting the expected quantitative values or qualitative goals for the evaluation
Defining a quality management plan	To define an action plan to monitor progress and quality of the project outputs	A plan for managing the quality of the project results was prepared as part of the project management activities. Bi-weekly calls and reporting among the partners were agreed upon

Table 2. Steps in the “Planning” phase of the DECI evaluation planning.

### How can eHealth evaluation planning guidelines be improved to support practice?

As evident from Fig. 1, the total number of steps (n = 13) in the DECI planning process was less than those in the GEP-HI (n = 52) and AHRQ (n = 18). These differences mainly arose from the different levels of aggregation of the steps. Mapping of the matches and no matches between the guideliens and the DECI case has been presented in Appendix 3.

### Analyzing phase

We started the analyzing phase with an exploration of the contexts and methodologies, and lessons learned in similar studies (step *Learning approaches from related studies*). We found this step helpful not only to understand the protocols of such studies better, but also to plan the evaluation such that it would increase the generalizability of DECI results and support learning across similar studies. However, the comparison with the guidelines revealed that neither of the guidelines emphasized the importance of such an activity. The GEP-HI suggests the exploration of the methods to be used based on the study purpose, objectives, study type, and the information needed (Step 1.8 in Appendix 1). However, screening of the related published work was not the focus of this step (or any other step). After the comparison, it was concluded that both guidelines provided no equivalent guidance related to the step *Learning approaches from related studies* of the DECI process.

Then, we aimed to gain an in-depth understanding of the context and the constraints of DECI in the step *Identifying constraints*. Several relevant steps were identified from the GEP-HI (Step 1.6 and 2.5 in Appendix 1). However, the GEP-HI suggests a descriptive approach and depicts the consideration of the constraints as a writing activity. The AHRQ recommends considering the impact of the context on the potential measures alone (Step H in Appendix 2), while in DECI, we found the constraints to be applicable to the data collection methods too (e.g., some methods may not be feasible to use on individuals with a particular diagnosis). Moreover, understanding the constraints was a social activity with the stakeholders, and every stakeholder had a complementary perspective and knowledge that allowed us to enrich the common understanding of the constraints and to plan the evaluation accordingly.

The step *Analyzing the feasibility of potential outcome measures* in DECI was an activity that involved the distribution of surveys among the project partners to identify their standard measurement practices (owing to the project’s interdisciplinary and multi-national nature). It also included multiple rounds of negotiations regarding which measures could be feasible and commonly acceptable, to improve the chances that the results of the evaluation would be used for decision-making and learning. Familiarity with the outcome measures was perceived as a contributing factor to the success of the evaluation. We assumed a non-directive approach and did not impose partners with a list of outcome measures to be used. Instead, we utilized a collaborative, consensus-based approach, where the partners sought for alignment on the measures to be used during the evaluation. The GEP-HI contains no equivalent steps; neither does it discuss the need to study the feasibility of using certain outcome measures, nor does it reflect upon different research settings and how certain steps should be approached in such cases. The AHRQ, on the other hand, is highly specific when it comes to the feasibility of the outcome measures (Step G, H, I, and J in Appendix 2) and reflects similar activities as those observed in the practice of DECI.

Then, we aimed to identify how the project partners approached the project and upcoming evaluation, through the step *Analyzing stakeholders perspectives*. A relevant step was identified in the GEP-HI (Step 2.3 in Appendix 1), which suggests the development of a descriptive map of the formal and informal organizational structures of an organization. Although we agree that such an activity is highly important, we believe that the GEP-HI did not advise its users to engage with the stakeholders, to discuss, and to gain insight on how they plan to approach the evaluation and the changes that may occur owing to the implementation of eHealth. In DECI, these activities were highly social and they were conducted through individual interviews and a group workshop with the stakeholders. This enabled us to derive a better understanding of the social structures and context. The AHRQ, on the other hand, suggests to consider what the team and the related stakeholders aim to gain from the evaluation, and what goals they carry (Step B in Appendix 2). However, the guideline does not specify how this understanding should be achieved.

The *Risk analysis* step in DECI involved discussions with all the partners in the project, and it revealed the differences between the risks identified by every partner. Differences in the line of business, competences, goals, and experiences led to diverse but complementary views on potential risks. The GEP-HI emphasizes the risk analysis step (Step 2.11 in Appendix 1), and depicts it as a descriptive activity, such as making a list of significant risks and defining a plan to counter them. The AHRQ does not reflect upon the need to perform a risk analysis.

### Planning phase

In the planning phase of the DECI process, the purposes and activities of the steps *Choosing a methodological approach*, *Defining evaluation questions*, and *Defining outcome measures* were well-addressed in the guidelines (see Appendix 3).

The steps *Planning data collection* and *Planning the monitoring of the data collection* in the DECI process were also recommended in the GEP-HI (Step 1.9, 2.8, 3.8, and 3.10 in Appendix 1) but not in the AHRQ. Here, our experience in DECI corresponds to a recommendation in the GEP-HI stating that the collection of data and its monitoring requires proper planning and consideration of ethical and legal aspects of privacy and data protection. Failure to set up the data collection according to such rules can jeopardize the evaluation and the use of its results. Moreover, monitoring of the data collection helps to ensure that the data collected is of desired quality.

Activities in the DECI step *Considering methods of data analysis* were also observed in the GEP-HI (Step 3.8 in Appendix 1) but not in the AHRQ. Our experience in DECI showed that this step can help to have a better understanding of (a) how the outcome measures will be used during the analysis, (b) whether all the outcome measures are needed for a meaningful analysis, (c) what competence is needed for the analysis, (d) how the plans for analysis align with the timeline and resources available, and (e) whether the analysis will be readable and understandable by the users of the evaluation results. Taking such matters into account helped us plan an early inclusion of the needed experts for specific analyses, to create more realistic expectations, and to define the scope of the analysis.

The *Defining expected results* step of DECI was recommended by the GEP-HI (Step 3.4 and 3.8 in Appendix 1) but not in the AHRQ. Our experience aligned with the recommendations in GEP-HI that, for every outcome measure, an expected result (or a frame of reference) can be established. This can be based on the experiences and goals of the stakeholders and on the related published work.

The *Defining a quality management plan* step of DECI was similar to Step 4.4 of the GEP-HI but it had no equivalent step in the AHRQ. The quality management plan in DECI was developed in response to the risk analysis performed. Monitoring of the identified risks and setting up the response measures were the final activities in the DECI evaluation planning.

**Fig. 2:**
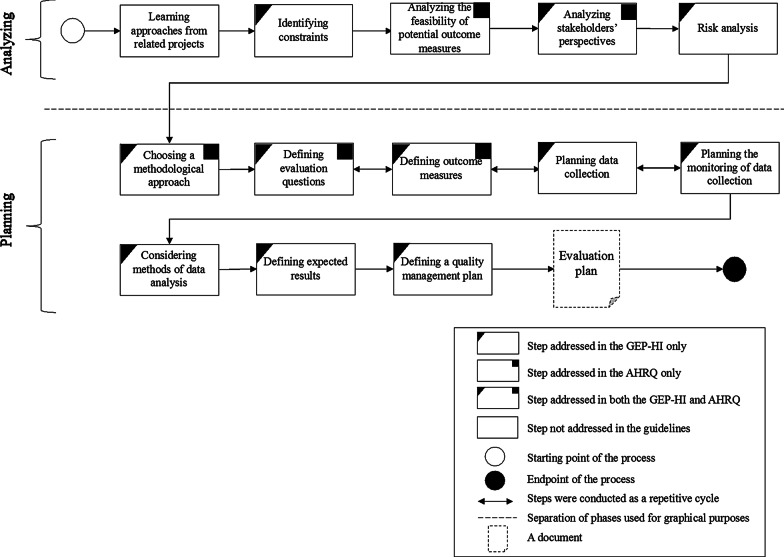
Comparison between the evaluation planning process of DECI and the GEP-HI and AHRQ guidelines

Figure 2 depicts the results of the comparison between the evaluation planning process of DECI and the GEP-HI and AHRQ guidelines.

## Discussion

The research objective of this study was to examine the eHealth evaluation planning process in a multi-national and interdisciplinary setting and to provide recommendations for the development of eHealth evaluation planning guidelines.

### eHealth evaluation planning process in a multi-national and interdisciplinary setting

The empirical process of eHealth evaluation planning demonstrated how the planning can be performed in a multi-national and interdisciplinary setting. Most of the planning activities required extensive negotiations and alignment of plans between the involved stakeholders, as the evaluation methodology had to be uniform for all the contexts. Ensuring use and sharing lessons learned from other similar projects, which is an important step for any program evaluation [13, 33], layed a strong foundation for these negotiations. To increase the willingness and competence of the stakeholders to use the evidence for decision-making and learning, we used a democratic collaborative approach during planning [34–36]. It helped to build consensus on key decisions, such as agreeing on a methodological approach and outcome measures. We identified that through the process of building consensus among stakeholders, the choice of approaches and measures gradually became more apt. This influenced the quality of the evaluation positively as inappropriate choice of methods and measures could be fatal for evaluation [5] which is a keybeen identified as major obstacle. Moreover, different agendas and methodological preferences to evaluation (e.g. medical research approach vs. quality improvement approach) could have jeopardized the ability to compare the evidence between different settings, in turn reducing the transferability of the whole study [15]. Moreover, a potential use of evidence created through evaluation could decrease if the stakeholders or decision-makers did not recognize the type of research conducted, did not understand or accept the methodologies or outcome measures, or if the quality of the evaluation was doubtful [37]. A drawback of this approach was an increased amount of time such an alignment demanded, as also observed in other contexts [14]. Therefore, agreeing to the proponents of planning (e.g., [22, 23]), we found that evaluation planning is important in larger set-ups that involve multi-national and interdisciplinary teams. Otherwise, individual stakeholders could benefit from the emergent approach to evaluation [10] that does not support planning in advance and promotes iterative testing and methodological adaptation based on the needs of a stakeholder.

### Propositions for improvement of eHealth evaluation planning guidelines

A comparison between the DECI process of evaluation planning and the guidelines for eHealth evaluation planning provided in the GEP-HI and AHRQ showed that, though these guidelines are useful for practice, they both have room for improvement. Our study showed that, in their present form, these guidelines may not be effective enough in preventing problems with the quality of the evaluation. For example, the AHRQ fails to address the monitoring of data quality, consideration of the laws on data protection and privacy, and general risk and quality management of the evaluation assignment and outputs. While ethical and legal aspects are considered as important topics for evaluation [15], bringing these issues at the planning stage is not sufficiently discussed in the current literature. Similarly, risk analysis of eHealth has been studied to an extent [9], whereas risk analysis as part of the evaluation process does not get sufficient attention. Additionally, our analysis showed that the GEP-HI provides an oversimplified view of the selection of outcome measures. During the selection, no feasibility analysis is recommended in the guideline, and the benefits of engaging the stakeholders who will use the evaluation results based on these measures are overlooked. Previous research has considered these activities imperative to ensure the quality of the evaluation [5, 6, 13, 38].

Both guidelines have overlooked the importance of encouraging users to screen the existing research in the subject area to identify the methods and outcome measures used, and to aim for methodological uniformity across different eHealth evaluation studies. Numerous scholars have identified the lack of methodological uniformity as a problematic area in eHealth evaluation studies, which affects the comparability of evidence and adoption of eHealth [4, 6–8, 39, 40]. Consideration of methodological approaches, measures, and lessons learned in similar evaluation studies can lead to more credible and generalizable results [3, 33, 41]. Moreover, methodological alignment between evaluation studies can promote the use of research evidence which has been lacking when making decisions for practice improvement [42, 43].

None of the guidelines examined in the present study provided guidance on how to identify, engage, and make use of the interdisciplinary or multi-national settings, nor did they provide any links to other guidelines addressing the same. This problem was also identified by Janssen, Hettinga, Visser, Menko, Prins et al. [40] in relation to the existing frameworks for the evaluation of eHealth. Moreover, the GEP-HI depicts several activities as writing or drawing activities carried out by an evaluator alone (steps such as identifying constraints, analyzing the stakeholders’ structures, and risk analysis). In the empirical DECI process, these activities required intense collaboration with the stakeholders. Our experience showed that complementary competences of the stakeholders can provide a wider spectrum of insight, which is in line with the findings of previous research [10, 11, 13]. With an increasing trend of working in interdisciplinary evaluation teams [8, 10], the guidelines need to evolve.

Academic community should be aware of the existing gap between methodologies and practice of eHealth evaluation. To reduce this gap, methodological materials developed by scholars should address the already reported quality issues [5–10] better. Academic community should also encourage more case reports like the one described in the present study, as these can help the scholarly discussions be more relevant to practice and prevent the common concerns of the quality issues in eHealth evaluations. In addition, scholars need to take variations in evaluation set-ups into consideration when discussing evaluation quality issues or proposing methodological material for evaluation. Different set-ups bring certain complexities in the evaluation process [10–14], especially during its planning, and it may impact the quality of evaluations and generalizability and comparison of the evidence across studies and contexts.

### Limitations and future research

The present study was limited to a single case. Although it can provide an in-depth scope for comparative analysis with existing eHealth evaluation guidelines, a multiple-case study might have provided a wider spectrum of evaluation planning practices in different set-ups. The analyzed case involved a multi-national and interdisciplinary team. However, there are other set-ups in which the evaluation planning process might differ, which means that the generalizability of the results and conclusions can be troublesome. On the other hand, a single case, like ours, can contribute to a theorethical generalization, i.e. the results and conclusions can be used to further develop theory on eHealth evaluation planning. Further, a limited number of eHealth evaluation planning guidelines were analyzed in this study. Reviewing other available guidelines can reveal different shortages in them and result in a different set of recommendations for improving the guidelines. Also, there might be a portion of e-mails and other internal communication documents not available to the authors affecting the data set in this study.

Future research should aim to identify the risks and strategies for quality associated to eHealth evaluation in different set-ups beyond single case studies, and to address those risks through evaluation planning activities.

## Conclusion

The extent and types of activities during planning of the evaluation can depend on the set-up of the evaluation team. Planning for evaluation assignments implemented by interdisciplinary and multi-national evaluation teams take more time for orchestration and control to ensure the quality of the evaluation. The standardized guidelines for evaluation planning can provide a great support to evaluators, if the guidelines address issues of evaluation quality more explicitly, and are updated with activities such as (1) analyzing the feasibility of outcome measures and data collection, based on the context and laws of data protection, (2) planning how to monitor the quality of the data, and (3) screening for methods and measures used in similar studies. The guidelines can also be complemented with strategies on how to benefit from, and overcome challenges connected to, different research set-ups.

## Data Availability

The datasets used and analyzed during this study are available from the corresponding author on reasonable request.

## Appendix 1: Numbering the steps of the GEP-HI

**Table:**

Phase	Items of the phase
1. Preliminary outline	1.1 Purpose of the study
	1.2 Primary audience
	1.3 Identification of the study-funding party(ies)
	1.4 First identification of stakeholders
	1.5 Identification of required expertise
	1.6 The organizational and user context of the evaluation study
	1.7 Object of evaluation, type of health IT
	1.8 First exploration of evaluation methods to be used
	1.9 Ethical and legal issues
	1.10 Budget
	1.11 Preliminary permissions for publication
	1.12 Result of preliminary outline
	1.13 Formal acceptance to proceed to the next phase
2. Study design	2.1 Detailed rationale and objectives of the study
	2.2 Key evaluation issues, questions, indicators
	2.3 Stakeholder analysis/Social network analysis
	2.4 Study methods
	2.5 Organizational context, the study setting
	2.6 Technical setting, the type of health IT
	2.7 Participants from the organization
	2.8 Project timeline
	2.9 Material and practical resources
	2.10 Establishment of the study team
	2.11 Risk analysis and quality management
	2.12 Budget
	2.13 Ethical and legal issues
	2.14 Strategy for reporting and disseminating the results
	2.15 Result of study design
	2.16 Formal acceptance to proceed to the next phase
3. Operationalization of methods	3.1 Study type
	3.2 Approach
	3.3 Assumptions and feasibility assessment
	3.4 Frame of reference
	3.5 Timing
	3.6 Justification of the methodological approach
	3.7 Expertise
	3.8 Outcome measures
	3.9 Avoiding bias
	3.10 Quality control on data (measures)
	3.11 Participants from the organization
	3.12 Ethical and legal issues
	3.13 Result of operationalization of methods
	3.14 Approval of operationalization of methods
4. Project planning	4.1 Project management
	4.2 Study flow
	4.3 Evaluation activity mapping
	4.4 Quality management
	4.5 Risk management
	4.6 Recruitment of necessary staff
	4.7 Inform all relevant stakeholders
	4.8 Result of project planning
	4.9 Approval of project planning

## Appendix 2: Numbering the steps of the AHRQ toolkit

**Table:**

Steps in AHRQ
A. Develop Brief Project Description
B. Determine Project Goals
C. Set Evaluation Goals
D. Choose Evaluation Measures
E. Consider Both Quantitative and Qualitative Measures
F. Consider Ongoing Evaluation of Barriers Facilitators, and Lessons Learned
G. Search for Other Easily Accessible Measures
H. Consider Project Impacts on Potential Measures
I. Rate Your Chosen Measures in Order of Importance to Your Stakeholders
J. Determine Which Measurements Are Feasible
K. Determine Your Sample Size
L. Rank Your Choices on Both Importance and Feasibility
M. Choose the Measures You Want to Evaluate
N. Determine Your Study Design
O. Consider the Impact of Study Design on Relative Cost and Feasibility
P. Choose Your Final Measures
Q. Draft Your Plan Around Each Measure
R. Write Your Evaluation Plan

## Appendix 3 Comparison of DECI process steps of planning an evaluation with GEP-HI and AHRQ toolkit

The column “Comparison” in Tables 3 and 4 presents matches between the steps of DECI and the steps of GEP-HI and AHRQ. The numbers denote the steps in GEP-HI (Appendix 1) and letters the steps in AHRQ (Appendix 2).

**Table 3:** Steps of the “Analyzing” phase of the DECI evaluation planning

Step	Comparison (matches with AHRQ and GEP-HI)
Learning approaches from related projects	GEP-HI: none AHRQ: none
Identifying constraints	GEP-HI: 1.6, 2.5 AHRQ: H
Analyzing the feasibility of potential outcome measures	GEP-HI: none AHRQ: G, H, I, J
Analyzing stakeholders’ perspectives	GEP-HI: 1.1, 1.4, 2.3 AHRQ: B
Risk analysis	GEP-HI: 2.11 AHRQ: none

**Table 4:** Steps of the “Planning” phase of the DECI evaluation planning

Step	Comparison (matches with AHRQ and GEP-HI)
Choosing a methodological approach	GEP-HI: 1.8, 3.1, 3.2, 3.3, 3.6 AHRQ: K, O, N
Defining evaluation questions	GEP-HI: 1.1, 2.1, 2.2 AHRQ: C
Defining outcome measures	GEP-HI: 2.2, 2.4, 3.8, 3.10 AHRQ: D, E, M, P, G, I, J, K, L
Planning data collection	GEP-HI: 1.9, 2.8, 3.8, 3.10 AHRQ: none
Planning the monitoring of data collection	GEP-HI: 3.10 AHRQ: none
Considering methods of data analysis	GEP-HI: 3.8 AHRQ: none
Defining expected results	GEP-HI: 3.4, 3.8 AHRQ: none
Defining a quality management plan	GEP-HI: 4.4 AHRQ: none

## Abbreviations

AHRQ: Health Information Technology Evaluation Toolkit
DECI: Digital Environment for Cognitive Inclusion
GEP-HI: Guideline for Good Evaluation Practice in Health Informatics
IMIA: International Medical Informatics Association
MAST: Model for Assessment of Telemedicine Applications
WHO: World Health Organization
